# Protein disorder in the human diseasome: unfoldomics of human genetic diseases

**DOI:** 10.1186/1471-2164-10-S1-S12

**Published:** 2009-07-07

**Authors:** Uros Midic, Christopher J Oldfield, A Keith Dunker, Zoran Obradovic, Vladimir N Uversky

**Affiliations:** 1 Center for Information Science and Technology, Temple University, Philadelphia, PA 19122, USA; 2 Center for Computational Biology and Bioinformatics, Indiana University School of Informatics, Indianapolis, IN 46202, USA; 3 Center for Computational Biology and Bioinformatics, Department of Biochemistry and Molecular Biology, Indiana University School of Medicine, Indianapolis, IN 46202, USA; 4 Institute for Intrinsically Disordered Protein Research, Indiana University School of Medicine, Indianapolis, IN 46202, USA; 5 Institute for Biological Instrumentation, Russian Academy of Sciences, 142290 Pushchino, Moscow Region, Russia

## Abstract

**Background:**

Intrinsically disordered proteins lack stable structure under physiological conditions, yet carry out many crucial biological functions, especially functions associated with regulation, recognition, signaling and control. Recently, human genetic diseases and related genes were organized into a bipartite graph (Goh KI, Cusick ME, Valle D, Childs B, Vidal M, et al. (2007) The human disease network. Proc Natl Acad Sci U S A 104: 8685–8690). This diseasome network revealed several significant features such as the common genetic origin of many diseases.

**Methods and findings:**

We analyzed the abundance of intrinsic disorder in these diseasome network proteins by means of several prediction algorithms, and we analyzed the functional repertoires of these proteins based on prior studies relating disorder to function. Our analyses revealed that (i) Intrinsic disorder is common in proteins associated with many human genetic diseases; (ii) Different disease classes vary in the IDP contents of their associated proteins; (iii) Molecular recognition features, which are relatively short loosely structured protein regions within mostly disordered sequences and which gain structure upon binding to partners, are common in the diseasome, and their abundance correlates with the intrinsic disorder level; (iv) Some disease classes have a significant fraction of genes affected by alternative splicing, and the alternatively spliced regions in the corresponding proteins are predicted to be highly disordered; and (v) Correlations were found among the various diseasome graph-related properties and intrinsic disorder.

**Conclusion:**

These observations provide the basis for the construction of the human-genetic-disease-associated unfoldome.

## Author summary

Many proteins with important biological functions lack stable structure under physiological conditions. These proteins, being known as intrinsically disordered, are very common in regulation, recognition, signaling and control, and play crucial roles in protein-protein interaction networks. Many of such intrinsically disordered proteins are associated with various human diseases such as cancer, cardiovascular disease, amyloidoses, neurodegenerative diseases, diabetes and others. Recently, human genetic diseases and related genes were organized into a specific network, diseasome. Previous analysis of this diseasome revealed several significant features including the common genetic origin of many diseases. However, the abundance of intrinsically disordered proteins involved in human genetic diseases and the functional repertoire of these proteins have never been before. We filled this gap by performing the thorough bioinformatics analysis of all the proteins form the diseasome utilizing several disorder predictors and by performing the intensive text mining. Here we show that intrinsic disorder is common in diseasome, and that proteins from different diseases possess different levels of intrinsic disorder. Many disordered regions are subjected to alternative splicing and contain specific molecular recognition features responsible for the protein-protein interactions. We also show that many hub proteins are generally more disordered than non-hub proteins. Our study provides the basis for the construction of the human-genetic-disease-associated unfoldome; i.e., a part of the diseasome dealing with the intrinsically disordered proteins.

## Introduction

Significant experimental and computational data show that many biologically active proteins lack rigid 3-D structure, remaining unstructured, or incompletely structured, under physiological conditions, and, thus, these proteins exist as dynamic ensembles of interconverting structures. These proteins are known by different names, including intrinsically disordered [1], natively denatured [2], natively unfolded [3], intrinsically unstructured [4], and natively disordered [5] among others. The terms intrinsic disorder (ID), intrinsically disordered protein (IDP), and intrinsically disordered region (IDR) will be used here.

The manifestation of ID is manifold, and functional disordered segments can be as short as only a few amino acid residues or can occupy rather long loop regions and/or protein ends. Proteins, even large ones, can be partially or even wholly disordered. Some IDPs and IDRs exhibit collapsed disordered conformations with pronounced residual structure (thus, resembling a molten globule), others can stay in extended highly disordered states (such as the random coil), while still others form collapsed random coils or semi-collapsed premolten globules [1,5-8]. The relationships among the different ID forms needs further study.

There are several crucial differences between amino acid sequences of IDPs/IDRs and structured globular proteins and domains. These differences include divergence in amino acid composition, sequence complexity, hydrophobicity, aromaticity, charge, flexibility index value, and type and rate of amino acid substitutions over evolutionary time. For example, IDPs are significantly depleted in bulky hydrophobic (Ile, Leu, and Val) and aromatic amino acid residues (Trp, Tyr, and Phe), which form and stabilize the hydrophobic cores of folded globular proteins. IDPs also possess a low content of Asn and of the cross-linking Cys residues. The residues that are less abundant in IDPs, and that are more abundant in structured proteins, have been called order-promoting amino acids. On the other hand, IDPs/IDRs are substantially enriched in polar and charged amino acids: Arg, Gln, Ser, Glu, and Lys and in structure-breaking Gly and Pro residues, collectively called disorder-promoting amino acid residues [1,9,10]. Thus, in addition to the well-known "protein folding code" stating that all the information necessary for a given protein to fold is encoded in its amino acid sequence [11], we have proposed that there exists a "protein non-folding code", according to which the propensity of a protein to stay intrinsically disordered is likewise encoded in its amino acid sequence [12,13].

Amino acid differences between IDPs and ordered proteins have been utilized to develop numerous disorder predictors, including PONDR^® ^(Predictor of Naturally Disordered Regions) [9], charge-hydropathy plots (CH-plots) [14] and IUPred [15] to name a few. Intrinsic disorder predictors fall into two general groups. Per-residue predictors (such as the PONDR^® ^group of predictors) output a score for each residue in a protein and are especially useful when applied to proteins having both structured and disordered regions. The other type of algorithm gives a single prediction value for the entire protein. This type is useful when the objective is to identify mostly or wholly disordered or structured proteins. The charge-hydrophobicity (CH)-plot and the cumulative distribution function (CDF) are the two main predictors of this type [16].

The current state of the art in the field of IDP predictions, including advantages and drawbacks, has been summarized recently [17]. Links to many of the servers for these predictors, when available, can be found in the Disordered Protein Database, DisProt [18].

Although experimentally characterized IDPs have been discussed in the literature over at least four decades, these proteins have not been viewed as a group but rather as a collection of unusual protein outliers. Bioinformatics is playing a major role in transforming this collection of examples into a sub-field of protein science. For example, soon after the first disorder predictor was developed [19], it was shown that 25% of proteins in Swiss-Prot contained predicted ID regions longer than 40 consecutive residues and that about 11% of residues in Swiss-Prot were likely to be disordered [20]. Subsequent analyses confirmed these trends and revealed that eukaryotic proteomes are significantly more enriched in IDPs in comparison to bacterial and archaeal proteomes [16,21]. This increased utilization of IDPs in higher organisms was attributed to the greater need for signaling and coordination among the various organelles in the more complex eukaryotic domain [1,22].

IDPs carry out numerous biological functions, many of which obviously rely on high flexibility and lack of stable structure. These functions are diverse and complement those of ordered proteins and protein regions. While structured proteins are mainly involved in molecular recognition leading to catalysis or transport, disordered proteins and regions are typically involved in signaling, recognition, regulation, and control by a diversity of mechanisms [23-25].

IDPs play crucial roles in protein-protein interaction networks, which generally involve a few proteins binding to many partners (called hub proteins or hubs) and many proteins interacting with just a few partners. Consideration of structure data revealed that several hub proteins are entirely disordered, from one end to the other, and to be capable of binding large numbers of partners, other hubs contain both ordered and disordered regions, and some hubs are structured throughout [26]. Fully disordered hubs can serve as scaffolds for organizing the components of multi-step pathways [27]. For the mixed-structure hubs, many, but not all, of the interactions map to the regions of disorder. For the highly structured hubs (such as 14-3-3 [28] and calmodulin [29]), the binding regions of their partner proteins are intrinsically disordered [30]. Overall, these observations support two previously proposed mechanisms by which ID is utilized in protein-protein interactions: namely, one disordered region binding to many partners and many disordered regions binding to one partner [30,31].

The binding diversity of IDPs plays important roles in the establishment, regulation and control of various signaling networks. Such disorder-based signaling is further modulated in multicellular eukaryotes by posttranslational modification and by alternative splicing, both of which very likely occur much more often in IDRs compared to structured regions of proteins [25,32]. Locating alternative splicing in disordered regions avoids the folding problems that arise upon removal of segments of from structured domains. The flexibility of IDRs facilitates the binding of the enzymes that bring about the disorder-associated posttranslational modifications. We have suggested that the intersection of binding sites, posttranslational modifications, and alternative splicing variants within IDRs provide a powerful combination to bring about signaling diversity in different cell types [25,28,32]

Many IDPs and IDRs fold upon binding with their specific partners. Said partners include other proteins, nucleic acids, membranes or small molecules [33]. The concept of the "molecular recognition feature," abbreviated as MoRF, was introduced to describe short, intrinsically disordered regions that "morph" from disorder-to-order upon partner recognition [34-36]. Based on several specific features in the disorder prediction scores, a predictor of helix-forming MoRFs was elaborated [34,37]. The application of this predictor to several proteomes revealed that such foldable recognition features are especially abundant among eukaryotic proteins [34,37]. MoRFs that form sheet or irregular structure also exist [35,36]. Predictors of these non-helical MoRFs have not yet been developed, so the predictions of helix-forming MoRFs should be regarded as providing lower-bound estimates of binding sites in disordered regions.

Proteins are involved in virtually all cellular and in many extracellular processes. Protein dysfunction can therefore cause development of various pathological conditions and a broad range of human diseases known as protein-conformation or protein-misfolding diseases. Such diseases arise from the failure of a specific peptide or protein to adopt its functional conformational state; i.e., from protein misfolding and malfunctioning.

Misfolding diseases can affect a single organ or be spread through multiple tissues. Consequences of misfolding include protein aggregation, loss of normal function, and gain of toxic function. Misfolding and misfunction can originate from point mutation(s) or result from an exposure to internal or external toxins, from impaired posttranslational modification (phosphorylation, advanced glycation, deamidation, racemization, etc.), from an increased probability of degradation, from impaired trafficking, from lost binding partners or from oxidative damage among other causes. These factors can act independently or in complex associations with one another [38]. Furthermore, numerous IDPs are associated with human diseases such as cancer [22], cardiovascular disease [39], amyloidoses [40], neurodegenerative diseases [41], diabetes and others [38]. Based on these intriguing links among intrinsic disorder, cell signaling and human diseases, suggesting that protein conformational diseases may result not only from protein misfolding, but also from misidentification and missignaling [30], the "disorder in disorders" or D^2 ^concept was recently introduced [38].

As a result of decades-long efforts, impressive lists of disease-gene association pairs were generated [42,43]. In parallel, analysis of protein-protein interactions in humans produced detailed maps of the relationships between different genes including those related to disease [44,45]. To gain a better understanding of the relationship between the genes implicated in a selected disease, network-based tools were successfully utilized for a single disease, e.g., human inherited ataxias and disorders of Purkinje cell degeneration [46].

Recently, to estimate whether human genetic diseases and the corresponding disease genes are related to each other at a higher level of cellular and organism organization, a bipartite graph was utilized in a dual way: to represent a network of genetic diseases, the "human disease network", HDN, where two diseases are directly linked if there is a gene that is directly related to both of them, and a network of disease genes, the "disease gene network", DGN, where two genes are directly linked if there is a disease to which they are both directly related [47]. This framework, called the human diseasome, systematically linked the human disease phenome (which includes all the human genetic diseases) with the human disease genome (which contains all the disease-related genes). This diseaseome opens a new avenue for the analysis and understanding of human genetic diseases, moving from single gene-single disease viewpoint to a framework-based approach [47].

The analysis of the HDN and DGN properties revealed that these networks are significantly different in many aspects from randomly generated networks of the same size. By these analyses the various diseases became classified into 20 types, some diseases were unclassified, and several diseases were annotated as belonging to multiple classes. Similarly, genes were clustered into classes via their associations with specific diseases [47]. Analysis of this network of genetic diseases and disease genes linked by known disease-gene associations revealed the common genetic origin of many diseases. The vast majority of these disease genes was non-essential and showed no tendency to encode hub proteins. Overall, the expression pattern of these disease-related genes indicated that they are localized in the functional periphery of the network [47].

In the present study, we started from the disease-related classification of genes from [47] and then performed a large-scale analysis of the abundance of intrinsic disorder in transcripts of the various disease-related genes. Since structural information was available only for a limited number of these proteins, we used intrinsic disorder predictions. We also analyzed the correlation between various HDN/DGN graph-related properties of genes and intrinsic disorder. We compared the occurrence of alternative splicing in various disease classes and analyzed the relationship between alternative splicing and intrinsic disorder. In essence, the aim of our study was to build an unfoldome, which we define as the IDP-containing subset of a given genome, associated with human genetic diseases.

Overall, our findings indicate that there are significant differences in occurrence of intrinsic disorder in the proteins arising from genes related to diseases as compared to proteins arising from genes unrelated to specific diseases. Furthermore, there are significant differences with respect to intrinsic disorder among the various disease classes. Our analysis shows noticeable positive trends that link intrinsic disorder to graph-related features of genes, such as the number of other genes that are directly linked to a given gene via the diseasome network. Certain disease classes have a significantly greater fraction of genes involved in alternative splicing, and these alternative splicing regions are predicted to be highly disordered. In summary, disorder analysis provides interesting new insights regarding the human diseasome.

## Methods

The basis for our experimental dataset is the dual Human Disease Network/Disease Gene Network (HDN/DGN) [47]. It consists of two types of nodes that represent human genes (1,777) and diseases (1,284), and links that connect diseases with related genes. A disease and a gene were connected by a link if mutation(s) in the corresponding gene were implicated in the given disease [47]. The network is dual, because it can be observed as both a Human Disease Network (two diseases are linked if they are both related to the same gene), or as a Disease Gene Network (two disease genes are linked if they are both related to the same disease).

We augmented the set of disease genes from DGN with human genes with known protein sequences. Protein sequences for all human genes were collected from NCBI Gene database; we excluded all model proteins obtained solely with automated genome annotation processing. After this exclusion, our dataset consists of 1,751 human disease related genes and 16,358 other human genes with known protein sequences. If several protein sequences were collected for a single gene; i.e., for genes with multiple alternatively spliced isoforms, then any duplicate sequences were discarded.

The diseases in DGN were grouped into twenty classes. In addition to these twenty classes we introduced sets of unclassified diseases and diseases belonging to multiple classes as two separate disease classes. We used this approach to classify genes as well. In our model, a gene belongs to all classes to which its related diseases also belong. Furthermore, since a gene can be related to multiple diseases that belong to various classes, we defined an additional *multiple class gene *group. Thus, overall, this approach defined 22 gene classes: the twenty original classes, as well as classes of *unclassified genes *(related to unclassified diseases) and *multi-class disease genes *(genes related to diseases that belong to multiple classes). Note that the 22 gene classes were not necessarily disjoint, and that all genes from *multiple class gene *class also belonged to at least two more classes. Two more sets were used for comparison: *disease genes *(this set included all genes from DGN with known protein sequences; i.e., genes from all 22 previously defined classes), and *human genes *(this was the whole dataset that included the *disease genes *set). Table 1 contains preliminary statistics for 22 disease/gene classes and 3 additional classes of genes, namely *multiple class genes*, *disease genes*, and *human genes*.

**Table 1:** Disease class names and acronyms, number of diseases and number of genes related to disease classes.

Class name	Acronym	Number of diseases	% (of 1284)	Number of genes	% (of 1751)
Skeletal	SKEL	64	4.98%	56	3.20%
Bone	BONE	30	2.34%	44	2.51%
Dermatological	DERM	48	3.74%	80	4.57%
Cancer	CANC	113	8.80%	207	11.82%
Developmental	DEVE	37	2.88%	53	3.03%
Multi-class disease	MCD	155	12.07%	209	11.94%
Cardiovascular	CARD	41	3.19%	96	5.48%
Muscular	MUSC	31	2.41%	68	3.88%
Immunological	IMMU	69	5.37%	115	6.57%
Ophthamological	OPHT	62	4.83%	120	6.85%
Connective tissue disorder	CTD	28	2.18%	51	2.91%
Endocrine	ENDO	56	4.36%	96	5.48%
Neurological	NEUR	117	9.11%	254	14.51%
Psychiatric	PSYC	17	1.32%	30	1.71%
Ear, Nose, Throat	ENT	6	0.47%	44	2.51%
Respiratory	RESP	13	1.01%	34	1.94%
Renal	RENA	36	2.80%	58	3.31%
Hematological	HEMA	88	6.85%	146	8.34%
Nutritional	NUTR	4	0.31%	22	1.26%
Gastrointestinal	GI	23	1.79%	34	1.94%
Unclassified	UNCL	31	2.41%	29	1.66%
Metabolic	META	215	16.74%	289	16.50%
Multiple class genes	MULT			295	16.85%
Disease genes	DIS			1751	100.00%
Human genes	HUM			18109	

The same order of classes is used in graphs in the Results section; the first 22 classes are sorted in descending order with respect to the median of disorder content (defined in Experimental procedures). The difference between "multi-class diseases" and "multiple class genes" is that "multi-class diseases" set includes genes that are only related to diseases that are classified as "multiple" in [47], whereas "multiple class genes" includes genes that are related to several diseases that belong to different classes.

### Intrinsic disorder prediction

Three predictors of intrinsic disorder were used on the protein sequences: PONDR^® ^VSL2B, CH and CDF. **VSL2B **is a variant of VSL2 predictor described in [48]. For an amino acid sequence, VSL2B outputs ID prediction in [0, 1] range per residue. These outputs were then compared to a threshold (we used the default threshold 0.5) and residues with prediction value greater than the threshold were predicted to be ID. In the case of multiple sequences for one gene, sequences were aligned using our own multiple alignment algorithm, which was aimed at rediscovering identical exons in multiple sequences by only matching identical amino acids and optimizing the alignment for long contiguous matched subsequences. A sequence obtained from such multiple alignments included all exons from individual sequences, and was considered to represent the whole gene sequence. For each position in the alignment sequence, we obtained a single prediction by averaging predictions for all residues from protein sequences that are aligned at that position.

CH and CDF give outputs that predict disorder on the level of whole proteins. The **CH **(Charge-Hydrophobicity) predictor is based on the finding [14] that two sets of proteins – a set of natively unfolded proteins and a set of small globular folded proteins – occupy two distinct regions in the charge-hydrophobicity phase space, and can be almost perfectly separated with a straight line. The CH predictor calculates the mean hydrophobicity and the mean net charge for a protein sequence, identifies the part of the charge-hydrophobicity plane that the corresponding point belongs to, and calculates its distance from the separating line. The **CDF **predictor [16,49] compiles the predictions of a per-residue predictor to a single binary predictor per protein, by observing the *cumulative distribution function *(CDF) of per-residue predictions, and comparing it to a set of 7 boundary CDF points obtained from a training set [16]. In the case of multiple sequences for one gene, we used weighted voting to determine a single prediction for the gene. For the CH predictor, we calculate the mean of signed distances (distance is multiplied by -1 if prediction is negative, i.e. protein is predicted to be ordered). The prediction for the gene depends on the sign of the weighted mean (disorder if the weighted mean is positive, order otherwise). Similarly to the CH predictor, CDF predictor has a parameter (*CDF count*), the mean of which over all proteins sequences for a gene is compared to the threshold to determine a single prediction for the gene.

Since VSL2B provides per-residue predictions, we measure the *disorder content*, which is defined as the fraction of residues in a protein sequence (or sequence alignment in case of alternative splicing) that is predicted to be disordered. This provides a single prediction value for a given gene. Note that, unlike the CH and CDF predictors, this prediction can take any value in the range [0, 1].

### α-MoRF predictions

The predictor of an α-helix forming Molecular Recognition Feature (α-MoRF) is based on observations that predictions of order in otherwise highly disordered proteins corresponds to protein regions that mediate interaction with other proteins or DNA. This predictor focuses on short binding regions within long regions of disorder that are likely to form helical structure upon binding [34]. It uses a stacked architecture, where PONDR^® ^VLXT is used to identify short predictions of order within long predictions of disorder and then a second level predictor determines whether the order prediction is likely to be a binding site based on attributes of both the predicted ordered region and the predicted surrounding disordered region. An α-MoRF prediction indicates the presence of a relatively short (~20 residues), loosely structured helical region within a largely disordered sequence [34]. Such regions gain functionality upon a disorder-to-helix transition induced by binding to partner sequences [35,36]. Recently it has been indicated that the α-MoRF predictor has a poor sensitivity, i.e., misses many α-MoRF regions [37], due to the small set of α-MoRF regions used in its development. In this study, the modified α-MoRF predictor, α-MoRF-PredII, was used [37]. This algorithm was improved by including additional α-MoRF examples and their cross species homologues in the positive training set, carefully extracting monomer structure chains from PDB as the negative training set and including attributes from recently developed disorder predictors, secondary structure predictions, and amino acid indices as attributes [37].

### Alternative splicing analysis

For genes with multiple isoforms, the multiple alignments provide the information on the alternative splicing regions. We define the alternative splicing regions (AS regions) as exons that are expressed in some, but not all protein sequences for a given gene. Similarly as for a whole gene, we define disorder content for an AS region as the fraction of its residues that are predicted to be disordered.

### Statistical analysis of the data

When disorder content measurements – as predicted by VSL2B predictor – for all genes in a disease class were observed as a sample, we used statistical tests to compare the samples arising from different disease classes. Since we cannot make any assumptions on the distributions for disorder content in disease classes, we used the nonparametric Mann-Whitney U test (Wilcoxon rank-sum test) [50,51] to test whether two samples of observations (i.e. disorder content for two classes) came from the same distribution. The Mann-Whitney U test was not appropriate for similar comparison in the case of CH and CDF predictors, as their predictions were binary. For these two predictors, we counted the number of positive (disordered) and negative (ordered) observation in two samples (classes) and then used the χ^2 ^test to estimate the likelihood of whether the two samples come from the same distribution.

We dealt with the possible problems of multiple hypotheses testing by controlling *false discovery rate *(FDR) with the Benjamini-Hochberg (for independent tests) [52] or with the Benjamini-Yekutieli method [53].

Several of our hypotheses dealt with the dependency between graph-related numeric properties of nodes representing genes and disorder content. The numeric properties were defined as:

• number of related diseases: number of diseases the gene is directly related to (as provided in [47])

• number of related disease classes: number of distinct disease classes that diseases related to the gene belong to

• degree: number of other genes that are related to the diseases the gene is related to; or defined in the terms of DGN graph: the number of other genes that are directly linked to the gene (through some disease node).

For such hypotheses we used (first-order) linear regression to model the relationship, and then we used the corresponding F-statistic to assess the validity of the linear model.

The HDN/DGN graph was not completely connected. Using the usual definition of connectivity in graphs, we identified the connected components. One of the components was large and included 516 disease nodes and 903 gene nodes. All of the remaining components contained 15 genes or less; for example, 399 components contain only one gene each. We split the set of disease genes (DIS) into the set of 896 disease genes that belong to the large component (LARGECOMP) and the set of 855 disease genes that belong to one of the smaller components (SMALLCOMPS). Note that although the 16 disease genes with no available protein sequences were not included in the DIS set, and therefore neither in LARGECOMP nor the SMALLCOMPS set, these 16 genes were still included in the HDN/DGN graph for the purpose of identification of connected components.

## Results

### Analysis of ID in human diseasome

Prediction of intrinsic disorder using PONDR^® ^VSL2B predictor on all 30053 initially collected protein sequences showed significant differences in predicted ID content for the 7525 (25.04%) model protein sequences obtained with automated genome annotation processing, and the 22528 (74.96%) protein sequences with additional experimental support. The medians of disorder content for model protein sequences was much higher (68.6% vs. 37.5%), as well as the first quartile (37.9% vs. 21.4%) and the third quartile (96.5% vs. 61.7%). Furthermore, 40.6% of model protein sequences were predicted to have disorder content above 80%, compared to only 11.3% for remaining sequences.

**Figure 1:**
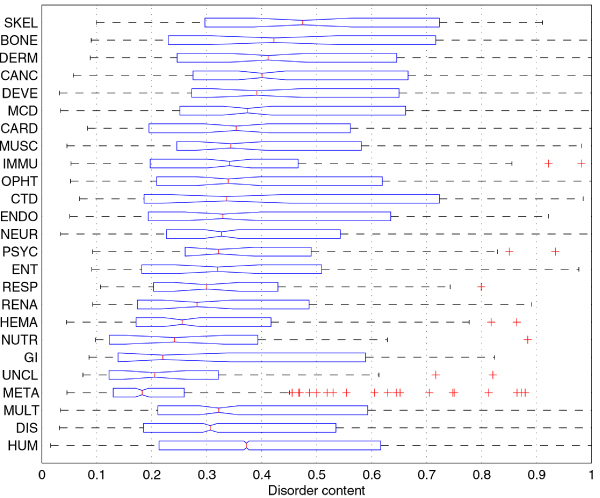
**Comparison of disorder content distributions in disease classes and human gene class using boxplots**. The 22 disease classes are sorted according to their disorder content medians. The boxes in the boxplot represent the first quartile (left edge), median (line in the middle), and third quartile (right edge); the whiskers extend to the lowest/highest values within the 1.5 IQR interval from the box (IQR is the range between the first and the third quartile), while the + signs represent the outliers. Medians for two classes can be compared by looking at the notches at their median lines; if the notches do not overlap, the medians are different at the significance level α = 0.05.

The boxplot in Figure 1 depicts the distributions of disorder content for genes in 25 classes. The 22 disease classes are sorted according to their medians of disorder content. The distributions for the majority of classes appear to be positively skewed. The ranges of disorder content between the first and the third quartile differ greatly between classes. For example, *connective tissue disorder *(CTD) class is ranked eleventh in disorder content median among the 22 disease classes, but has the highest third quartile.

**Figure 2:**
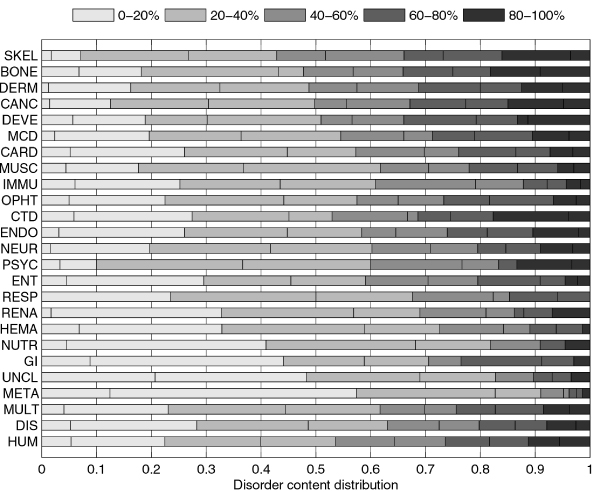
**Comparison of disorder content distributions in disease classes and human gene class using stacked histograms**. The histograms are stacked horizontally to save space. They show what fraction of genes in each class has disorder content within various ranges. Each of the five major ranges, that cover 20% each, is further split into two smaller 10% ranges (they use the same color, but are divided with a line). Distributions can be visually compared by observing the balance between darker and lighter shades of gray; the class with a darker histogram has on average more disorder content.

The distributions of disorder content in disease classes are further compared in histograms in Figure 2. The various classes have irregular disorder content distributions that can hardly be fit by any of the standard distributions. Furthermore, the distributions associated with the different disease classes are dissimilar both in shape and size. For these reasons we use a nonparametric test, Wilcoxon rank-sum test [50,51], to compare the distributions by comparing their medians.

**Figure 3:**
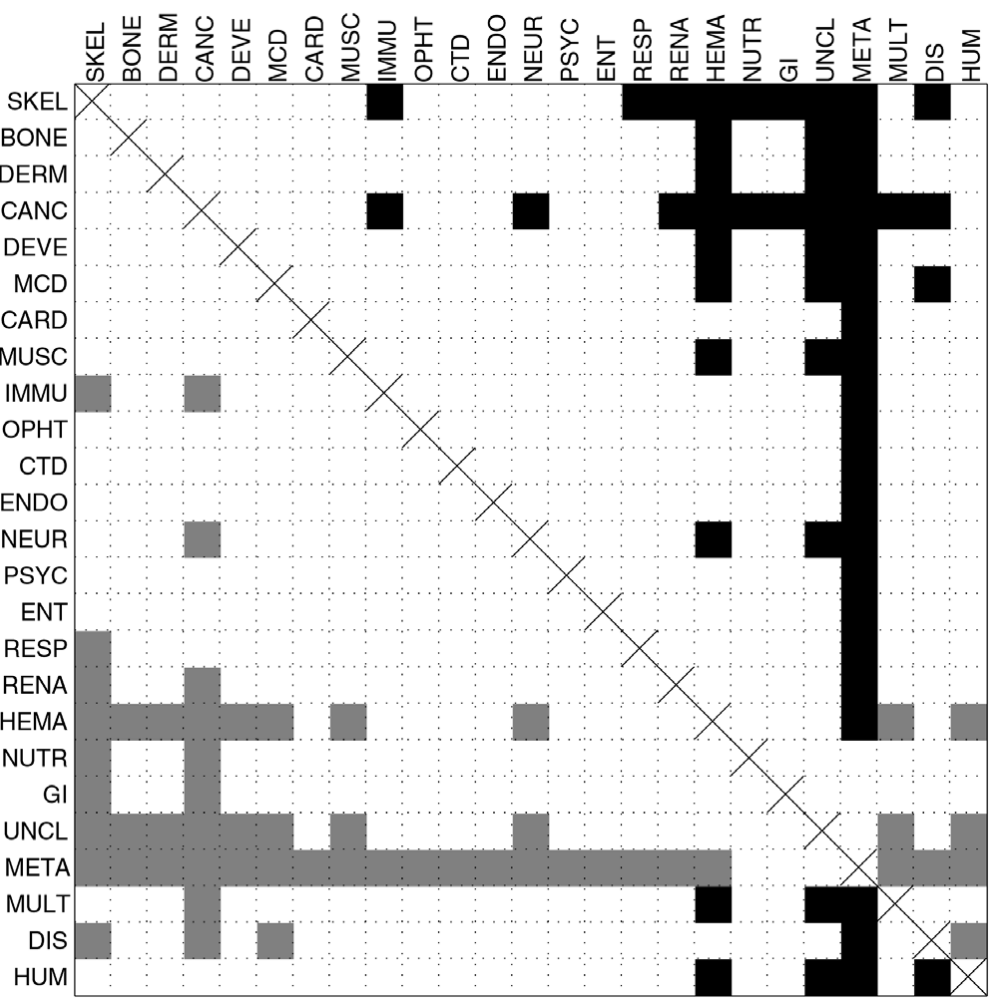
**Pairwise comparison of disorder content medians for disease classes and human gene class**. Filled squares represent pairs for which adjusted Wilcoxon rank sum test *p*-values are smaller than α = 0.05 (*p*-values are adjusted for false discovery rate control with Benjamini-Yekutieli method). Squares are filled black if the median for the row class is greater than the median for the column class, or gray if the median for the row class is smaller than the median for the column class.

Figure 3 shows an overview of pair-wise comparisons of disorder content medians. We used Benjamini-Yekutieli (BY) method of *false discovery rate *(FDR) control [53], as the *family-wise error rate *multiple comparisons methods, such as the Tukey-Kramer method [54,55], are much more conservative. With an FDR rate of 0.05, it is expected that 2.8 of 56 class pairs reported to have significantly different disorder content medians were false discoveries. The BY method is still quite conservative as it does not make any assumption on the independence of the pair-wise comparisons. Therefore we included Table 2 which shows the top 15 *p*-values and BY adjusted *p*-values for comparison of disease classes with *disease gene *(DIS) set, as well as for comparison of disease classes with *human gene *(HUM) set. Several other classes, besides the one indicated in Figure 2, can be considered to have disorder content medians significantly different from the DIS and HUM classes, depending on how strict the comparisons are to be. For example, *cancer gene *class has (borderline) significantly different disorder content median than the *human gene *set with a BY false discovery rate of 0.05. Several other classes have low p-values in comparison with *human gene *set, but the adjustment for the BY method pushes them above the 0.05 limit. Note that adjusted *p*-values would be ~3.7 times smaller if we used the Benjamini-Hochberg false discovery method [52], which makes an assumption that the tests are independent.

**Table 2:** Comparison of disorder content medians of disease classes with disease gene set (DIS) and with human gene set (HUM).

Comparison with DIS			Comparison with HUM		
	*p*-value	BY *p*-value		*p*-value	BY *p*-value

META	9.10·10^-31^	7.81·10^-29^	META	1.38·10^-50^	1.25·10^-48^
CANC	9.76·10^-09^	4.19·10^-07^	DIS	6.16·10^-15^	2.79·10^-13^
SKEL	3.92·10^-05^	0.001123	HEMA	7.13·10^-08^	2.15·10^-06^
MCD	0.000548	0.011771	UNCL	0.000192	0.004349
DERM	0.001852	0.031810	CANC	0.002397	0.043445
HEMA	0.003684	0.052740	NUTR	0.007141	0.107855
UNCL	0.004152	0.050941	SKEL	0.011080	0.143441
DEVE	0.008386	0.090036	GI	0.015816	0.179167
NEUR	0.033455	0.319267	IMMU	0.016768	0.168843
BONE	0.042742	0.367102	RENA	0.026136	0.236856
NUTR	0.063282	0.494113	RESP	0.093824	0.772967
MULT	0.090375	0.646849	MULT	0.105644	0.797813
MUSC	0.122463	0.809091	DERM	0.178919	1.247247
GI	0.130811	0.802516	ENT	0.195823	1.267578
ENDO	0.164391	0.941288	DEVE	0.208293	1.258409

Both the p-values and the adjusted p-values (for Benjamini-Yekutieli FDR control method) are listed in the table.

**Figure 4:**
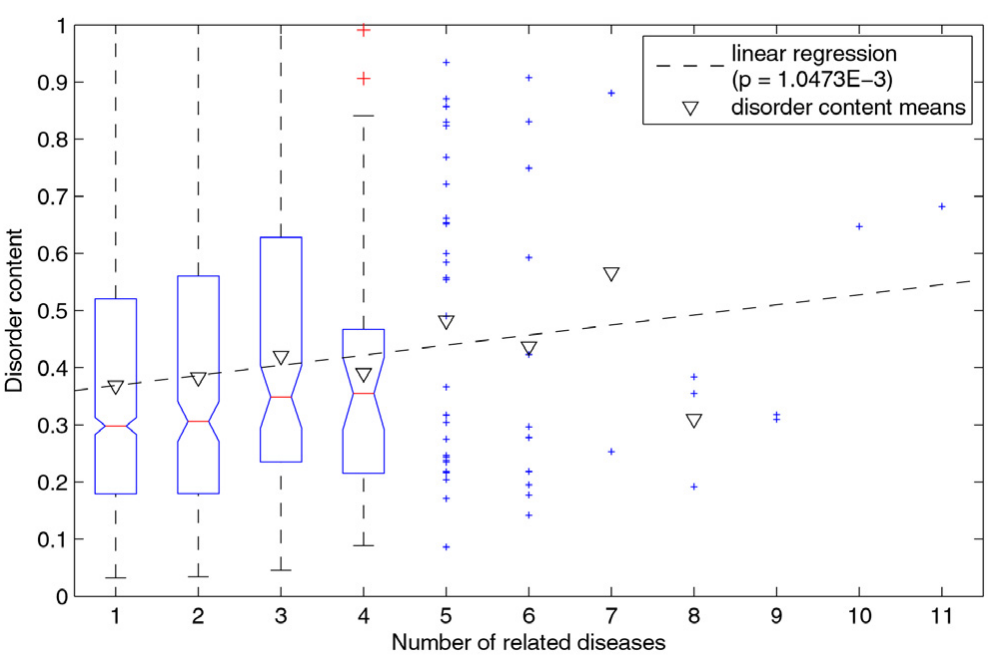
**Linear regression of disorder content with respect to number of related diseases (for genes)**. The genes with number of related diseases up to 4 are represented as a boxplot, while the remaining genes are represented as points. Note that the disorder content means (inverted triangles) for subsets are greater than the respective medians, because the disorder content distributions in these subsets are positively skewed.

**Figure 5:**
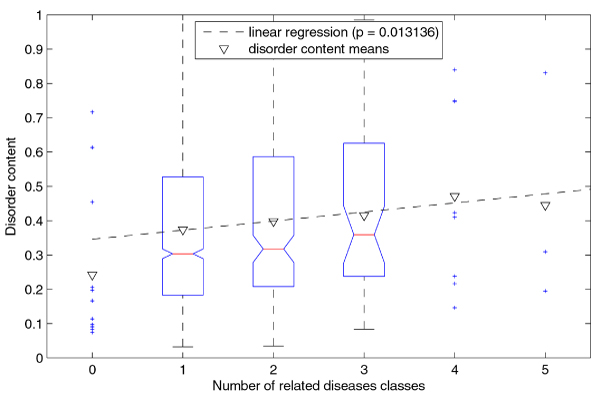
**Linear regression of disorder content with respect to number of related disease classes (for genes)**. The genes with number of related disease classes between 1 and 3 are represented as a boxplot, while the remaining genes are represented as points. Note that the disorder content means (inverted triangles) for subsets are greater than the respective medians, because the disorder content distributions in these subsets are positively skewed.

**Figure 6:**
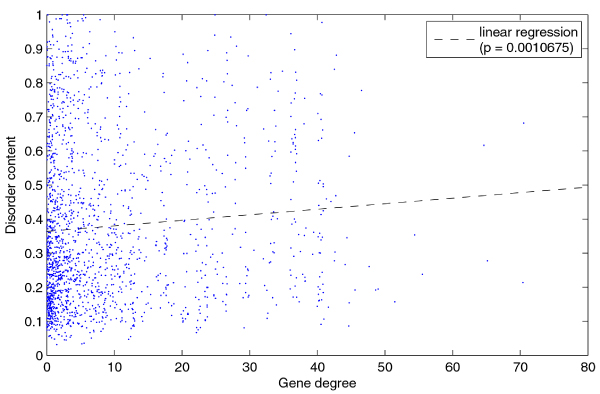
**Linear regression of disorder content with respect to gene degree in DGN**.

We continued with the investigation of the relationship between disorder content and several HDN/DGN graph-related properties. We used linear regression to model disorder content as a linear function of *number of related diseases for a gene *(Figure 4), *number of related disease classes for a gene *(Figure 5), and *gene degree in DGN *(Figure 6). For all three cases, the F-test gave *p*-values that were smaller than 0.05; for the *number of related diseases *and *gene degree *the *p*-values were smaller than 0.01. Although it is not likely that the observed linear trends were obtained by pure chance, they explained only a very small amount of variation in the disorder content; the respective R^2 ^values were 6.12·10^-3^, 3.51·10^-3^, and 6.10·10^-3^.

**Figure 7:**
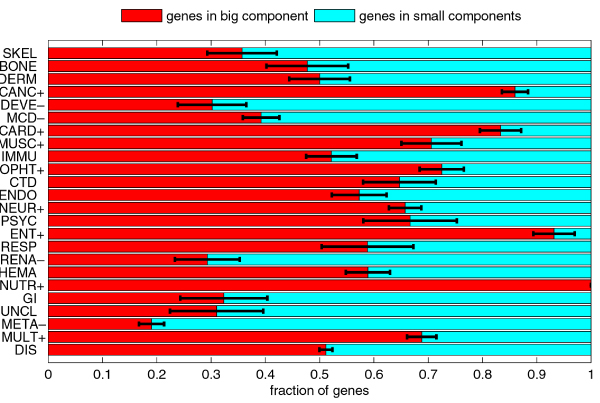
**Comparison of fractions of disease genes in the large component and the small components of the DGN**. The classes with the + signs after their acronyms are significantly overrepresented in the big component; the classes with the – signs after their acronyms are significantly underrepresented in the big component. The error bars represent one standard deviation or 68.2% confidence interval.

**Figure 8:**
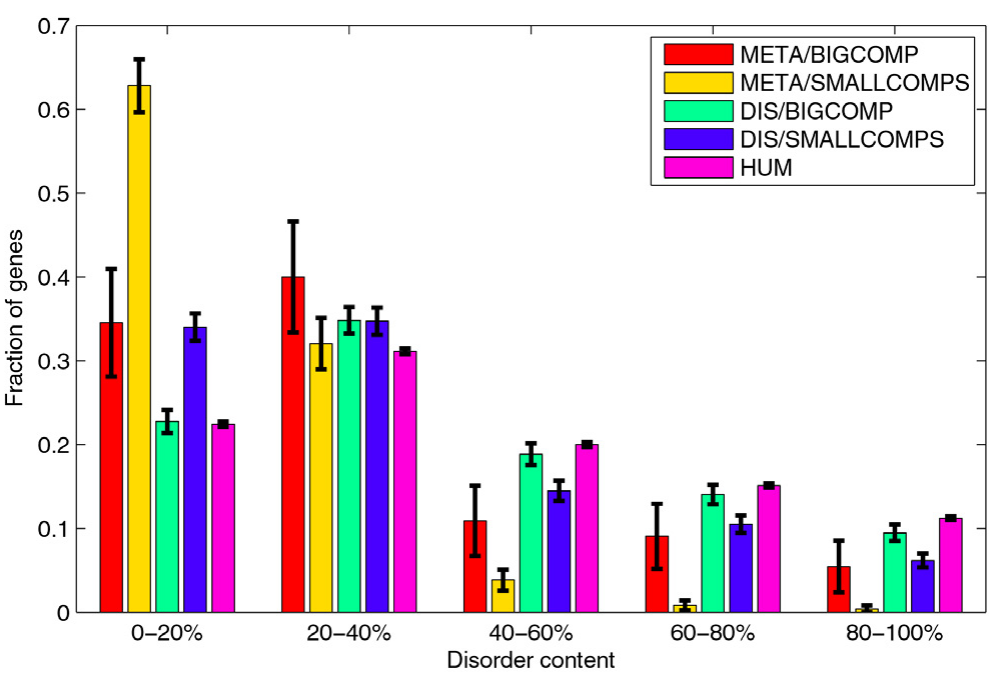
**Comparison of distributions of disorder content in LARGECOMP and SMALLCOMP for genes related to metabolic diseases, and for the whole disease gene set**. Distribution of disorder content for human gene set is included for comparison. The error bars represent one standard deviation or 68.2% confidence interval.

The disease genes set DIS is split almost evenly between LARGECOMP, the 896 (51.17%) disease genes in the large DGN component, and SMALLCOMPS, the 855 (48.83%) disease genes in the remaining small DGN components. This split can be further observed in individual disease classes. The histogram in Figure 7 shows the split between LARGECOMP and SMALLCOMPS for all disease gene classes. Using the χ^2 ^test to compare the split in each class to the overall split in the disease gene set, we identified classes of disease genes that were significantly overrepresented or underrepresented in LARGECOMP. For example, 85.99% of genes related to cancer diseases belonged to the large component, while only 19.03% of genes related to metabolic diseases belonged to the large component. We then compared the medians of disorder content for genes from LARGECOMP and SMALLCOMPS for each class individually, as well as for the whole disease genes set. The median of disorder content for LARGECOMP genes was significantly greater than for SMALLCOMPS genes, with an adjusted *p*-value of 7.56·10^-7 ^on the rank sum test. Similarly, the median of disorder content for LARGECOMP genes related to metabolic diseases was significantly greater than for the SMALLCOMPS genes related to metabolic diseases, with an adjusted *p*-value of 0.0112. These comparisons are illustrated in Figure 8. Substantial differences between disorder content medians for genes in LARGECOMP and genes in SMALLCOMPS can also be observed for several other classes; in the majority of cases, the median for the LARGECOMP genes is greater than the median for the SMALLCOMPS genes. However, none of these differences were statistically significant; which was partially due to the small numbers of genes in subsets compared.

### Alternative splicing and ID in human diseasome

**Figure 9:**
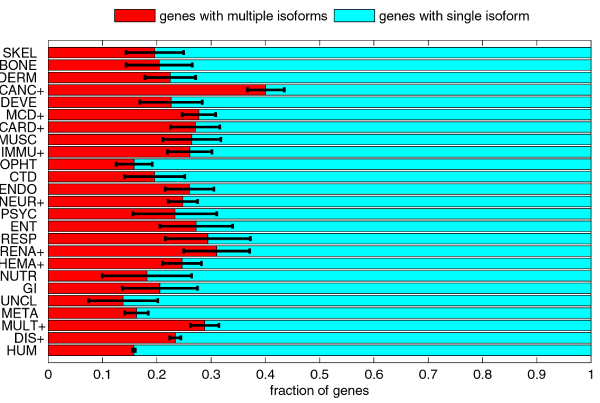
**Comparison of fractions of disease genes with multiple isoforms (i.e. with alternative splicing) and with single isoform**. The classes with the + signs after their acronyms have significantly higher fraction of genes with multiple isoforms than the human gene set. The error bars represent one standard deviation or 68.2% confidence interval.

We applied similar methodology to analyze alternative splicing. We divided the set of all genes (HUM) into the set of genes with multiple isoforms and the set of genes with a single isoform. The same division can also be applied to all disease classes, and the disease gene set. The comparison of fractions of genes with multiple isoforms is shown in Figure 9.

The disease gene set DIS had significantly higher fraction of genes with multiple isoforms than the human gene set HUM. Out of 1751 disease related genes, 410 genes (23.4%) had multiple isoforms (average of 2.77 for disease related genes with multiple isoforms), and they included 991 alternatively spliced regions (2.41 AS regions per disease-related gene with multiple isoforms). Out of 16358 non-disease genes, 2445 (14.95%) had multiple isoforms (average of 2.51 for non-disease genes with multiple isoforms), and they included 4954 AS regions (2.02 AS regions per non-disease gene with multiple isoforms).

Furthermore, all the disease classes but one (unclassified diseases) had higher fraction of genes with multiple isoforms than the HUM set, and for several classes this difference in fractions was statistically significant. The highest fraction of genes with multiple isoforms was 40.10% for the cancer disease gene class.

The comparisons of distributions of disorder content for genes with multiple isoforms with genes with single isoform showed that for three sets the medians of disorder content for genes with multiple isoforms were significantly greater than for genes with single isoform: human genes set HUM (BY adjusted *p *= 1.50·10^-7^), disease genes set DIS (BY adjusted *p *= 5.08·10^-7^) and multiple class genes set MULT (adjusted *p *= 0.0176). Individual tests for three disease classes also returned low *p*-values (hematological, *p *= 0.0196; renal, *p *= 0.0283; bone, *p *= 0.0291), but the corresponding BY adjusted *p*-values were above α = 0.05.

**Figure 10:**
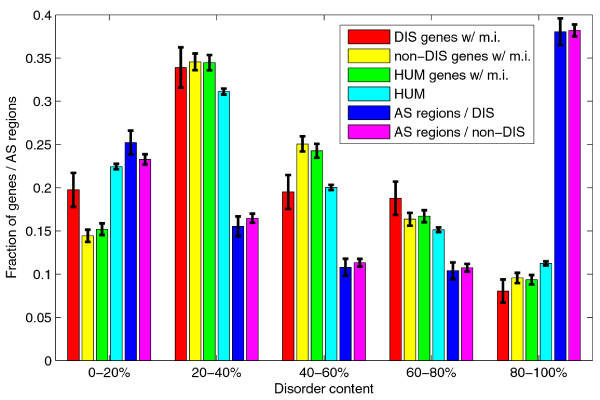
**Comparison of disorder content distributions for whole proteins and for alternative splicing (AS) regions**. Series #1–4 represent disorder content distributions in 1) disease genes with multiple isoforms, 2) non-disease genes with multiple isoforms, 3) human genes with multiple isoforms, 4) all human genes. Series #5 and #6 represent disorder content distributions for AS regions in disease genes, and AS regions in non-disease genes. The error bars represent one standard deviation or 68.2% confidence interval.

**Figure 11:**
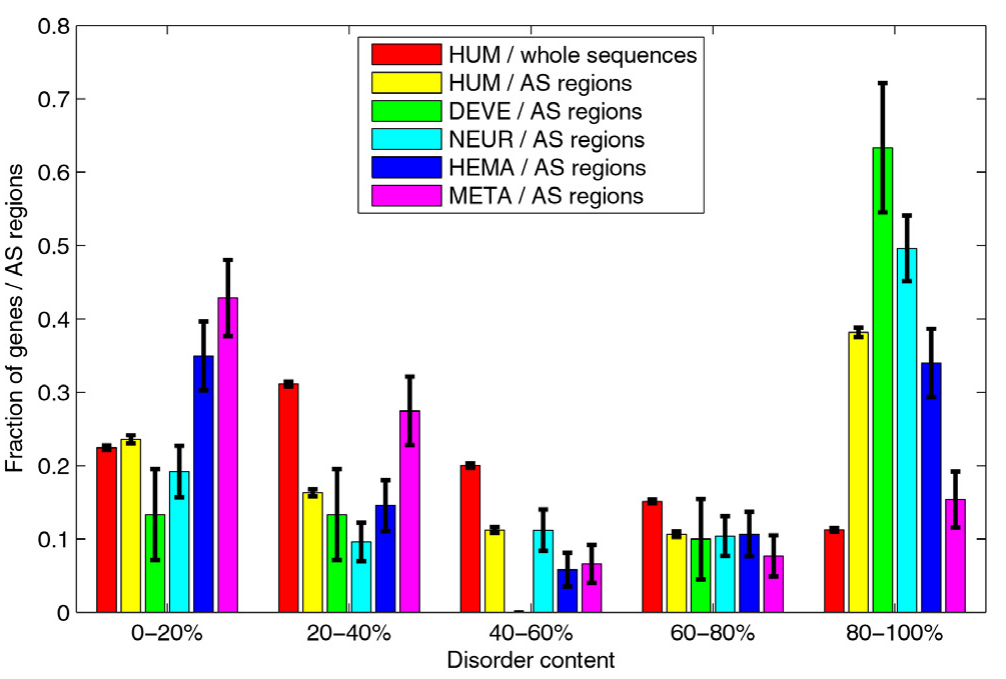
**Comparison of disorder content distributions for AS regions in various classes of human genes**. Series #1 and #2 represent disorder content distribution for whole human gene sequences and AS regions in human genes. Series #3–6 represent disorder content distributions for AS regions in: 3) developmental, 4) neurological, 5) hematological, and 6) metabolic disease classes. The error bars represent one standard deviation or 68.2% confidence interval.

Figure 10 shows the distributions of disorder content for genes with multiple isoforms (disease, non-disease, and all genes) and for all human genes. Although there are significant differences in medians, the distributions have similar shapes; the peaks are in the 20–40% range, and the fractions decrease with the increase in the disorder content. Figure 10 also shows the disorder content distributions for AS regions in disease related and non-disease genes. These two distributions have different shape than shape of the disorder content distributions for whole proteins; the fractions decrease with the increase in the disorder content, but then suddenly increase in the 80–100% range. The rank sum test for medians shows that the distribution of disorder content in AS regions is significantly different from distributions of disorder content in whole proteins for all genes (*p *~10^-142^), as well as for subset of genes with multiple isoforms (*p *~10^-48^). However, as is clearly seen in Figure 10, the distributions of disorder content for AS regions in disease genes and non-disease genes were not significantly different (*p *= 0.5278). We compared the disorder content distributions for AS regions for genes from individual classes to the overall distribution for AS regions from all human genes. The distributions for classes with significant statistical results are shown in Figure 11. For developmental and neurological disease classes, the fraction of AS regions in 80–100% range is significantly increased. Similarly, there is an increase in 0–20% range for hematological disease class. Metabolic disease class is an extreme case, as there is both a big increase in 0–20% range and decrease in 80–100% range; the AS regions in metabolic disease genes have significantly less disorder when compared to whole sequences in human genes.

### α-MoRFs in the human diseasome

**Figure 12:**
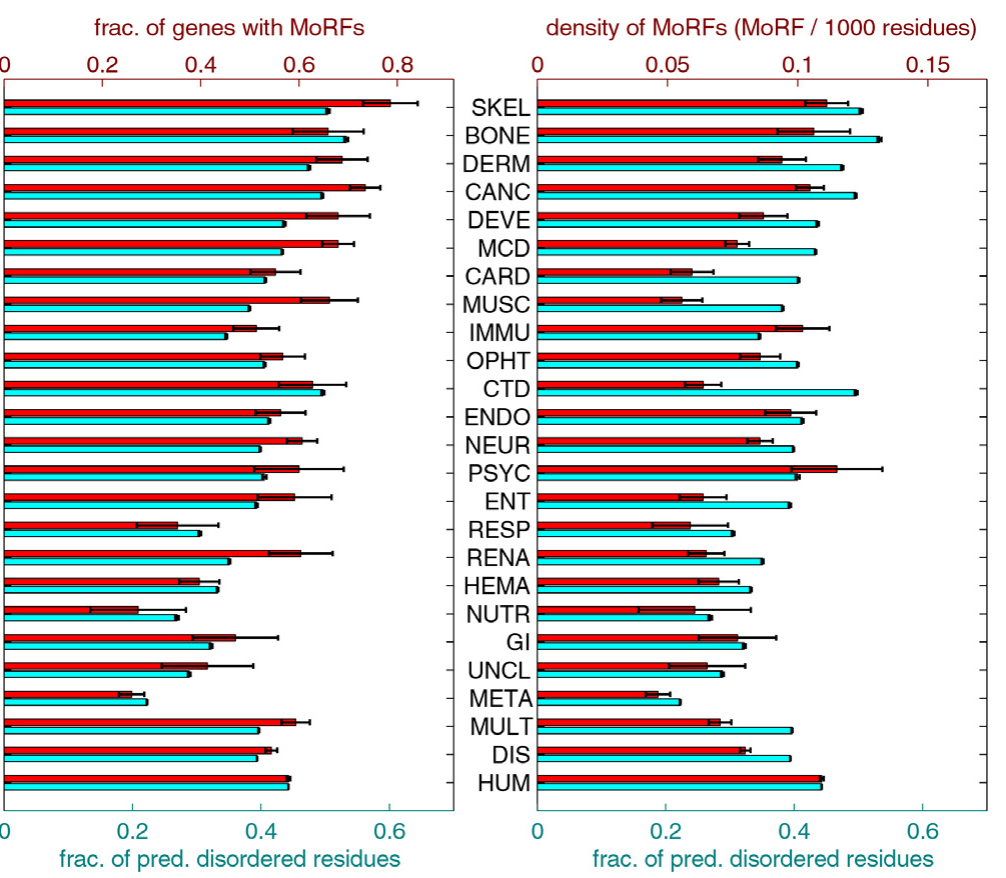
**Comparison of fractions of genes with predicted α-MoRFs and densities of α-MoRFs with fractions of disordered residues**. The plot on the left compares fractions of genes with predicted α-MoRFs (top/first series) with fractions of disordered residues (bottom/second series). The plot on the right compares densities of α-MoRFs (top/first series) with fractions of disordered residues (bottom/second series). In both plots the series are shown with different scales, such that the values for HUM set are aligned. The error bars represent one standard deviation or 68.2% confidence interval.

Figure 12 compiles the α-MoRF prediction data and shows the fractions of genes with predicted α-MoRFs and the densities α-MoRFs (number of α-MoRFs per residue) for all disease classes, as well as for sets of all disease genes and all human genes. The overall fractions of disordered residues are included for comparison. The fractions of genes with predicted α-MoRFs are highly correlated to fractions of disordered residues (corr. coefficient ~0.89).

### α-MoRFs and alternative splicing in the human diseasome

**Figure 13:**
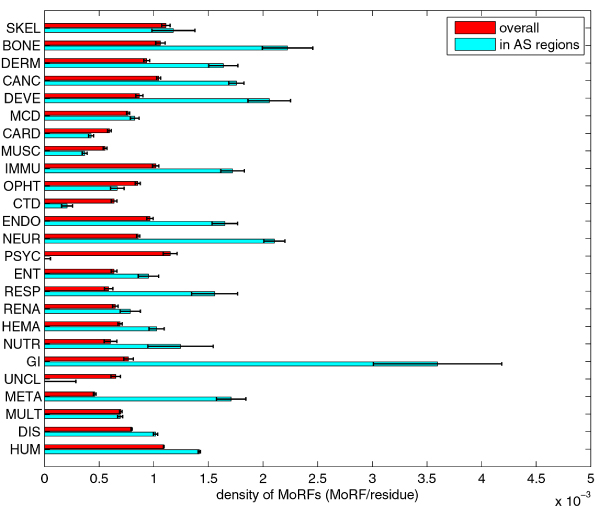
**Comparison of overall density of predicted MoRFs vs density of predicted MoRFs in AS regions for 25 classes/sets**. The error bars represent one standard deviation or 68.2% confidence interval.

Figure 13 compares the overall density of predicted α-MoRFs versus density of predicted α-MoRFs in AS regions for the 25 classes. The differences between densities of MoRFs (overall vs. AS regions) are significant for the majority of classes (listed by increasing *p*-values: HUM, NEUR, META, CANC, DIS, GI, DEVE, IMMU, ENDO, RESP, BONE, DERM, MUSC, CARD, HEMA, ENT), borderline significant for NUTR and OPHT, and not significant for the remaining classes (RENA, MCD, UNCL, SKEL, MULT). Two classes (PSYC, UNCL) have no α-MoRFs predicted in AS regions (while genes in both classes have very small number of residues in AS regions, for PSYC class this difference in densities is still statistically significant). Finally, Table 3 lists the quotients of the MoRF density in AS regions over the overall MoRF density, as well as corresponding *p*-value for comparison of these densities.

**Table 3:** Comparison of densities of predicted α-MoRFs in AS regions and complete genes.

Class acronym	Density of MoRFs in AS/Overall density of MoRFs	p-value for comparison of densities of MoRFs
GI	4.68	3.84·10^-021^
META	3.71	1.16·10^-061^
RESP	2.65	1.19·10^-010^
NEUR	2.45	2.54·10^-076^
DEVE	2.37	4.76·10^-017^
BONE	2.10	1.28·10^-010^
NUTR	2.06	0.017694
DERM	1.75	5.11·10^-010^
ENDO	1.71	2.93·10^-011^
IMMU	1.69	1.92·10^-013^
CANC	1.68	1.07·10^-031^
ENT	1.50	0.00079422
HEMA	1.48	4.65·10^-007^
HUM	1.30	6.12·10^-233^
DIS	1.27	1.10·10^-030^
RENA	1.21	0.55174
MCD	1.08	0.55638
SKEL	1.06	2.8735
MULT	0.99	3.0621
OPHT	0.78	0.049566
CARD	0.72	3.30·10^-007^
MUSC	0.66	8.89·10^-009^
CTD	0.32	3.78·10^-006^
PSYC	0	2.07·10^-005^
UNCL	0	0.56375

The quotients of density of predicted α-MoRFs in AS regions over overall density of predicted α-MoRFs and the *p*-values for comparison of corresponding densities.

### Evaluation of ID by binary classifiers

**Figure 14:**
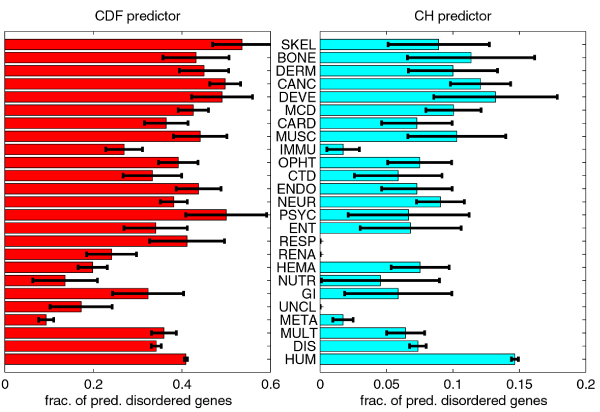
**Fractions of genes predicted to be disordered by CDF and CH predictors**. The error bars represent one standard deviation or 68.2% confidence interval.

We compared the fractions of genes predicted to be disordered by per-protein predictors CDF and CH in Figure 14. Overall, the CDF predictor identified more genes to be disordered than the CH predictor. The ratio was 2.79 for human gene set, and 4.64 for the disease genes set. For disease classes it ranged from 2.63 for hematological disorder genes to 15.50 for immunological disorder genes; additionally, for three disease classes – respiratory, renal and unclassified – the CH predictor predicted all genes to be ordered. The correlation coefficient for two vectors of fractions was 0.66.

When compared to HUM gene set, the CDF predictor identified significantly different fractions of disorder for the classes META, DIS, HEMA, and (borderline significance) IMMU, while the CH predictor predicted significantly different fractions of disorder for the classes DIS, META, MULT, IMMU, and RENA (in all these classes, fractions of predicted disordered genes were significantly smaller than the fraction for HUM set). When compared to DIS gene set, the CDF predictor identified significantly different fractions of disorder for classes META, CANC, HEMA, and SKEL, while the CH predictor predicted significantly different fractions of disorder for class META. Classes META and HEMA had smaller fractions of predicted disordered genes than DIS set, and classes CANC and SKEL had greater fractions of predicted disordered genes than DIS set.

The relationship between alternative splicing and intrinsic disorder, as predicted by CDF and CH predictors, can only be observed at the level of whole proteins. For the CDF predictor, classes with significantly different fractions of predicted disordered genes in genes with single isoform and in genes with multiple isoforms were: DIS, HUM, RENA, MULT, and (borderline significance) HEMA; in all cases, fraction of predicted disordered genes for genes with multiple isoforms was greater than for genes with a single isoform. For the CH predictor, significant difference of fractions of predicted disordered genes in genes with a single isoform and in genes with multiple isoforms was only observed in the HUM set.

In general, CDF predicts a much higher fraction of genes to be disordered than CH. Vectors of fractions of predicted disordered genes in various classes for CDF and CH predictors are fairly correlated, though there are several classes with substantial differences. For example, IMMU, RESP, RENA, and UNCL have very low (or even zero) fractions of disordered genes for CH predictor. The relative difference between HUM and DIS sets is much larger for the CH predictor (approximately two-fold) than for the CDF predictor. For the CDF predictor, the fractions of predicted disordered genes for several classes are higher than (although not strictly significantly higher) or similar to the same fraction in the HUM set, while this is not the case for CH predictor.

Overall, the fractions of predicted disordered genes for both binary predictors are correlated to medians of disorder content for VSL2B predictors, but there are some striking differences. For example, low fractions of disordered genes in IMMU class for both predictors, or relatively high fraction of PSYC and RESP classes for CDF predictor. Looking only at the medians without at least comparing whole distributions is not a good way to compare prevalence of intrinsic disorder in two classes/sets of genes.

The difference between these two methods in the magnitude of predicted disorder is generally similar to previously published data [16,39,56]. This difference was explaine by the fact that the CH-plot is a linear classifier that takes into account only two parameters of the particular sequence – charge and hydropathy, whereas CDF analysis is dependent upon the output of the PONDR^® ^VLXT predictor, a nonlinear neural network classifier, which was trained to distinguish order and disorder based on a significantly larger feature space that explicitly includes net charge and hydropathy. According to these methodological differences, CH-plot analysis is predisposed to discriminate proteins with substantial amounts of extended disorder (random coils and pre-molten globules) from proteins with globular conformations (molten globule-like and rigid well-structured proteins). On the other hand, PONDR-based CDF analysis may discriminate all disordered conformations including molten globules from rigid well-folded proteins. Therefore, this discrepancy in the disorder prediction by CDF and CH-plot might provide a computational tool to discriminate proteins with extended disorder from native molten globules, which might be predicted to be disordered by CDF, but compact by CH-plot. This model is consistent with the behavior of several intrinsically disordered proteins (e.g., [57]).

**Figure 15:**
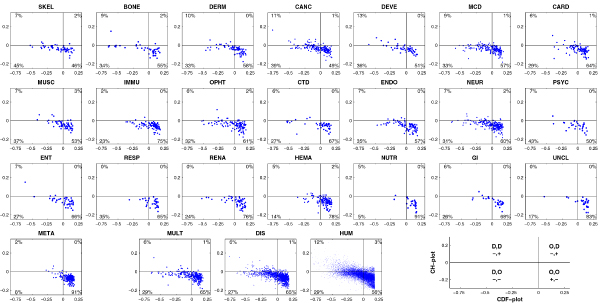
**Comparison of CDF and CH predictions in various disease gene classes and gene sets**. Each spot represents a gene whose coordinates were calculated as the distance of the corresponding point in the CH-plot from the boundary (x-coordinate) and the averaged distance of the corresponding CDF-curve from the CDF boundary (y-coordinate). Four quadrants in each plot correspond to the following predictions: (-,-) proteins predicted to be disordered by CDF, but compact by CH, (-,+) proteins predicted to be disordered by both methods, (+,-) ordered proteins, (+,+) proteins predicted to be disordered by CH, but ordered by CDF. This is further illustrated by an explanatory plot at the bottom right corner. Percentages represent the fractions of genes in the corresponding quadrants.

Figure 15 compares the results of the CH-plot and CDF analyses by showing the distributions of proteins in each disease within the CH-CDF phase space. In these plots, each spot corresponds to a single protein and its coordinates are calculated as a distance of this protein from the boundary in the corresponding CH-plot (Y-coordinate) and an averaged distance of the corresponding CDF curve from the boundary (X-coordinate). Positive and negative Y values correspond to proteins which, according to CH-plot analysis, are predicted to be natively unfolded or compact, respectively. Whereas positive and negative X values are attributed to proteins that, by the CDF analysis, are predicted to be ordered or intrinsically disordered, respectively. Therefore, each plot contains four quadrants: (-, -) contains proteins predicted to be disordered by CDF, but compact by CH-plot (i.e., potential native molten globules); (-, +) includes proteins predicted to be disordered by both methods (i.e., proteins with extended disorder); (+, -) contains ordered proteins; (+, +) includes proteins predicted to be disordered by CH-plot, but ordered by the CDF analysis. A sharp cut-off at the right side of each plot is due to the upper limit of a difference between the CDF curve (which might have a maximum value of 1.0) and a boundary separating IDPs and ordered proteins in CDF plots. Figure 15 suggests that the majority of the wholly disordered proteins could possibly be native molten globules.

## Discussion

### Intrinsic disorder in predicted and experimentally identified proteins

The difference in predicted disorder content between sets of predicted model protein sequences and (partially) confirmed protein sequences is greater in both magnitude and statistical significance than difference between any two classes in our final data set. The simplest explanation for this is that automated annotation procedure has a high error rate that introduces a large number of incorrect amino acid sequences. Alternatively, this dramatic difference in the level of predicted ID between the experimentally and automatically identified proteins could be due the bias of the existing identification techniques toward the ordered proteins. To some extent this resembles a problem the Structural Genomics Initiative Centers are facing, where the use of the traditional target search criteria (mostly based on the sequence identity) and protein purification and isolation methods generated mostly ordered targets, whereas alternatively identified and purified proteins awaiting structure determination were richer in disorder than an average protein in PDB [58,59]. It has been pointed out that this bottleneck was determined by the strategy chosen were in efforts to identify proteins with novel folds researchers started with proteins having amino acid sequences unlike those of proteins with known 3D structures [58,59]. In a similar manner, traditional experimental approaches developed for protein identification could be biased toward order (as ordered well-folded proteins where at the research focus for many years), whereas predictive tools are mostly dealing with the remaining part of the proteomes and therefore are inevitably identifying more disordered proteins.

The predicted sequences were unevenly distributed between disease-related and disease-unrelated proteins. In fact, the majority of the predicted "model" sequences were products of the non-disease genes. Therefore, including such sequences into the data set would introduce significant bias for disorder in the non-disease gene part of the data set. Based on these observations, we decided to exclude such sequences from the final datasets.

An important assumption that we made was that ID predictors have no bias towards any class of genes. Although errors are unavoidable in prediction of disorder, we assumed that both false positive and false negative errors occur equally likely in all gene classes. Under this assumption we can expect that any observed variations in predicted disorder content between disease classes are due to real variations in disorder content and not due to bias introduced by prediction. Although we have not found any obvious reason for questioning these assumptions, more structural data are needed to test such biases.

### Intrinsic disorder in human genetic diseases

Contrary to our initial expectations based on known abundance of ID in such diseases as cancer [22], cardiovascular disease [39], amyloidoses [40], neurodegenerative diseases [41], diabetes and others [38], the disease genes have in general slightly lower disorder content than the non-disease genes. This can be explained by the fact that the human disease network (HDN) and disease gene network (DGN) are based on the genetic diseases and genes, mutations in which were associated with disease development, respectively. Based on the expression pattern analyses of the DGN genes it has been concluded that they are mostly localized in the functional periphery of the protein-protein interaction network [47]. This peripheral localization of most disease genes was explained assuming that mutations in topologically central, highly connected, and widely expressed genes were more likely to result in severe impairment of normal development, leading to early lethality and therefore to deletion from the population, whereas mutations compatible with survival into the reproductive years were more likely to be maintained in a population [47]. Overall, the vast majority of disease genes in DGN was non-essential and showed no tendency to encode hub proteins [47]. On the other hand, the above mentioned studies on various individual diseases [22,38-41] dealt with all proteins known to be associated with a given disease and not just those proteins bearing the disease-promoting mutations. Therefore, the various datasets of proteins associated with individual diseases contained wider variety of proteins, including hubs. It is important to remember that hub proteins were shown to be highly enriched in intrinsic disorder [26-28,60-64]. In fact, hubs were shown to have multiple interactions, either being intrinsically disordered and serving as an anchor, or acting as a stable globular scaffold that interacts with intrinsically disordered regions of its targets [26-28,60-64]. Therefore a systematic depletion of hub proteins in HDN and DGN can in part explain their slightly lower disorder contents.

### Functional analysis of the disorder predictions for several specific diseases

Our data revealed that there was a large variation in disorder content in various disease classes. Several disease classes had median disorder content higher or comparable with the human gene set HUM (SKEL, BONE, DERM, CANC, DEVE, MCD), and/or several classes had a higher or comparable fraction of highly disordered genes (SKEL, BONE, CTD, DERM, DEVE, CTD, PSYC, MCD).

Unfortunately, structural information on proteins associated with various genetic diseases is sparse. Therefore, in the analyses given below we used an established earlier correlation between various protein functions and ID [23-25]. The strongest correlation with ID was observed for regulatory functions involved in biological processes such as those described by the following functional keywords: differentiation, transcription, transcription regulation, spermatogenesis, DNA condensation, cell cycle, mRNA processing, mRNA splicing, mitosis, apoptosis, protein transport, meiosis, cell division, Ubl conjugation pathway, Wnt signaling pathway, chromosome partition, neurogenesis, ribosome biogenesis, chondrogenesis, growth regulation [23-25]. The major ID-associated functional keywords covered a wide spectrum of protein activities including ribonucleoprotein, ribosomal protein, developmental protein, chromatin regulator, hormone, growth factor, GTPase activation, cytokine, GAP protein, repressor, cyclin, activator protein phosphatase inhibitor.

Many of these processes and functions are performed by proteins associated with various human genetic diseases. Based on this correlation between ID and protein function, a given protein was assigned either to the ordered protein family or to the IDR class, assuming that if this protein possesses ID-associated function then it likely contains at least one long IDR. A brief overview of ID-enriched classes of human genetic diseases is presented below. In addition, several examples of disease-related genes encoding for important well-characterized IDPs are discussed. This includes disordered proteins, mutations in which were associated with particular diseases, α-synuclein (one of the major players in the Parkinson's diseases pathogenesis), p53 (a key tumor-supressor protein), huntingtin (a protein involved in the Huntington's disease pathogenesis), BRCA1 (a breast and/or ovarian cancer-associated protein) and EWS-FLI1 fusion protein (a protein associated with the Ewing's sarcoma family of tumors).

### Illustrative examples of well-studied IDPs encoded by disease-related genes

**α-Synuclein **is a typical IDP that links various synucleinopathies, a group of neurodegenerative disorders characterized by deposition of aggregated α-synuclein in the cytoplasm of selective populations of neurons and glia [65-68]. Clinically, synucleinopathies are characterized by a chronic and progressive decline in motor, cognitive, behavioral, and autonomic functions, depending on the distribution of the lesions. Some of the most common synucleinopathies include Parkinson's disease, dementia with Lewy bodies, Alzheimer's disease, Down's syndrome, multiple system atrophy, and neurodegeneration with brain iron accumulation type 1. Different diseases are characterized by the morphologically different α-synuclein-containing inclusions. In Parkinson's diseases and various Lewy body diseases these inclusions are Lewy bodies and Lewy; multiple system atrophy is characterized by the accumulation of glial cytoplasmic inclusions and neuronal cytoplasmic inclusions; whereas axonal spheroids are frequently found in neurodegeneration with brain iron accumulation type 1 [69,70].

Several observations implicate α-synuclein in the pathogenesis the pathogenesis of Parkinson's disease. For example, a direct role for α-synuclein in the neurodegenerative processes in PD and Lewy body dementia was demonstrated by genetic evidence. Autosomal dominant early-onset Parkinson's disease and Lewy body dementia was shown to be induced in a small number of kindreds as a result of three different missense mutations in the α-synuclein gene, corresponding to A30P, E46K, and A53T substitutions in α-synuclein [71-73] or as a result of the hyper-expression of the wild type α-synuclein protein due to gene triplication [74-77]. Besides this genetic evidence many other observations correlate α-synuclein and PD pathogenesis (reviewed in [67-70,78-82]. Some of these observations are briefly outlined below. The recombinant α-synuclein easily assembles into amyloid-like fibrils *in vitro *and this process is modulated by familial point mutations. Characteristic depositions in various synucleinopathies invariably contain aggregated α-synuclein. α-Synuclein is abnormally phosphorylated, ubiquitinated, and nitrated in pathology-related inclusions. Co-expression of chaperones or β-synuclein with α-synuclein in transgenic animals was shown to suppress the neurodegeneration. α-Synuclein-positive proteinaceous deposits were shown to accumulate in several animal models where Parkinsonism was induced by exposure to different neurotoxicants. All this indicates that α-synuclein is a key player in the pathogenesis of several neurodegenerative disorders

Conformational behavior of α-synuclein under a variety of environments has been extensively analyzed (for recent reviews see [69,70,82]). This analysis has revealed that the structure of α-synuclein is extremely sensitive to the environment and can be easily modified. As a result, α-synuclein was shown to possess a remarkable conformational plasticity, being able to adopt structurally unrelated conformations including the substantially unfolded state, an amyloidogenic partially folded conformation, different α-helical or β-structural species folded to a different degree, both monomeric and oligomeric, several morphologically different types of aggregates, including various oligomers, amorphous aggregates, and amyloid-like fibrils [69,70,82]. Based on this astonishing conformational behavior the concept of a protein-chameleon was proposed, according to which the structure of α-synuclein to a dramatic degree depends on the environment: the choice between all the mentioned above conformations is determined by the peculiarities of protein surroundings [82]. Functionally, α-synuclein is an example of disordered hub, as in a case-by-case studies, this protein was shown to interact with at least 50 ligands and other proteins [78], whereas a recent proteomic analysis identified 587 proteins involved in the formation of complexes with α-synuclein in the dopaminergic cells, with 141 proteins displaying significant changes in their relative abundance (increase or decrease) after these cell were treated with rotenone [83].

**Huntingtin **is a large protein with an estimated molecular mass of 350 kDa, which contains a polyglutamine tract near its N terminus expansion of which causes Huntington's disease [84]. Huntington's disease is a member of the family of neurodegenerative diseases associated with the expansion of a CAG repeat in the gene which is translated into the extension of the polyglutamine (polyQ) tract in the corresponding protein. The polyQ repeat varies between 16 and 37 residues in healthy individuals, and individuals who are afflicted by disease have repeats of >38 residues. The mechanistic hypothesis linking CAG repeat expansion to toxicity involves the tendency of longer polyQ sequences, regardless of protein context, to form insoluble aggregates [85-93]. The far-UV CD spectra of polyQ peptides with various repeat lengths were shown to be nearly identical and were consistent with a high degree of random coil structure, suggesting that the length-dependence of disease is not related to a conformational change in the monomeric states of expanded polyQ sequences [92]. In contrast, there was a dramatic acceleration in the spontaneous formation of ordered, amyloid-like aggregates for poly(Gln) peptides with repeat lengths of greater than 37 residues.

The N terminus of wild-type huntingtin interacts with proteins involved in nuclear functions, including HYPA/FBP-11, which functions in pre-mRNA processing (splicesome function) [94], nuclear receptor co-repressor protein (NCoR) [95], which plays a role in the repression of gene activity, and p53 [96], a tumor suppressor involved in regulation of the cell cycle. Full-length huntingtin contains candidate binding sites for other proteins with nuclear functions. Huntingtin contains a P*X*DLS motif, a candidate-binding site for the transcriptional corepressor C-terminal binding protein (CtBP) [97], suggesting that huntingtin may play a role in transcriptional repression.

The **p53 protein **is a transcription factor that targets genes involved in cell cycle regulation and apoptosis, among other functions [98]. p53 is at the center of a large signaling network, regulating expression of genes involved in such cellular processes as cell cycle progression, apoptosis induction, DNA repair, response to cellular stress, etc. [99]. When p53 function is lost, either directly through mutations or indirectly through several other mechanisms, the cell often undergoes cancerous transformation [100]. In fact, it is believed that all human cancers exhibit defects in the p53-signaling pathway [101]. p53 is considered as the most commonly mutated tumour-suppressor gene in human cancers [102]. In roughly half of all cancer cases the p53 gene is mutated [100]. Typically these are missense mutations within the DNA-binding core domain resulting in the expression of a protein with aberrant function. Among missense mutations, there are hotspot mutations at four codons (175, 248, 249 and 273), which together account for over 25% of all missense mutations identified in human cancers [103]. Cancers showing mutations in p53 are found in colon, lung, esophagus, breast, liver, brain, reticuloendothelial tissues and hemopoietic tissues [100]. For these reasons, a loss of p53 function is believed to be a major factor in cancer development [100] and this protein has attracted significant attention of cancer researchers. A database of p53 point mutations was created , which currently is the largest single-locus mutation database, containing more than 10,000 somatic mutations identified by sequencing [103].

There are three structural domains in p53: N-terminal translational activation domain, central DNA binding domain, and C-terminal tetramerization and regulatory domain. The analysis of the intrinsic order-disorder state in these revealed that the DNA binding domain is intrinsically structured, whereas the terminal domains are intrinsically disordered [104,105]. It has been shown that p53 induces or inhibits over 150 genes, including *p21*, *GADD45*, *MDM2*, *IGFBP3*, and *BAX *[106]. At the transactivation region, p53 interacts with TFIID, TFIIH, Mdm2, RPA, CBP/p300 and CSN5/Jab1 [99]. At the C-terminal domain, it interacts with GSK3β, PARP-1, TAF1, TRRAP, hGcn5, TAF, 14-3-3, S100B(ββ) and many other proteins [99]. Overall, ~70% of the interactions between p53 and its binding partners are mediated by IDRs in p53 [28]. A bias toward intrinsic disorder is even more pronounced in the sites of posttranslational modifications, with 86%, 90%, and 100% of observed acetylation, phosphorylation, and protein conjugation sites, respectively, found in IDRs [28]. This concentration of functional elements within IRDs comparing to just 29% of the residues being disordered [26,28] clearly shows that p53 extensively utilizes IDRs to mediate and modulate interactions with other proteins.

#### BRCA1

About 5%–10% of breast cancer and ovarian cancer are hereditary and 30%–50% of these are due to the autosomal dominant mutations in the susceptibility genes, *BRCA1 *and *BRCA2 *[107]. In both cases the variants are distributed uniformly along the entire coding region and intronic sequences flanking each exon [108]. Women with the *BRCA1 *mutations are susceptible to the development of a breast cancer before age 35–40 and of an ovarian cancer with a probability rate of, respectively, 45%–60% and 20%–40%. Women carrying *BRCA2 *mutations present a 25%–40% risk of breast cancer development and a 10%–20% risk of an ovarian cancer development [108].

BRCA1 participates in many different cellular pathways, including transcription, apoptosis and DNA repair, through direct or indirect interaction with a variety of partners [109]. It has multiple alternatively spliced isoforms. One of the most studied BRCA1 isoform has 1863 amino acids and comprises a long highly disordered central region flanked by ordered domains at the two termini. At the N-terminus is a RING finger domain of 103 residues. This domain is reported to form a heterodimer with BARD1 (BRCA1 associated RING domain 1) and to bind to the ubiquitin carboxy-terminal hydrolase BAP1. At the C-terminus are two tandem copies of the BRCA1 C-terminal domain (BRCT) with 218 total residues for the two domains. These two domains are reported to bind with transcriptional activators and repressors like CtlP.

The structural characterization by various spectroscopic techniques revealed that the 1500 amino acid long central region of BRCA1 is completely disordered [110]. However this disordered central region contains molecular recognition domains for both DNA and several protein binding partners, including tumor suppressors such as p53, retinoblastoma protein (RB) and BRCA2; oncogenes like c-Myc and JunB; DNA damage repair proteins such as Rad50 and Rad51; and the Fanconi anemia protein (FANCA) [110]. Importantly, BRCA1 was shown to have at least 24 alternatively spliced isoforms [111]. Alternative splicing was shown to affect mostly central IDR of BRCA1 modulating its functionality by removing different functional domains [32].

#### EWS-FLI1 fusion protein

Ewing's sarcoma family of tumors is a set of highly malignant tumors of bone and soft tissue that occur in children, adolescents, and young adults. These tumors share a recurrent and specific t(11;22) (q24;q12) chromosome translocation [112], which combines the N-terminus of EWS (residues 1–264) from chromosome 22 with the C-terminus of FLI1 (232 carboxy-terminal residues) from chromosome 11 to form EWS-FLI1 fusion protein, a chimeric transcription factor. EWS-FLI1 is expressed only in tumor cells and its function is required for the malignant phenotype of Ewing's sarcoma family of tumors [113]. EWS-FLI1 retains the Ets DNA binding domain from FLI1 and modulates a diverse group of target genes by binding to specific promoters including transforming growth factor-β receptor type-II [114], p21 (WAF1/CIP1) [115], PTPL1 [116], Id2 [117], andtenascin-C [118], EAT-2 [119], mE2C [120], manic fringe [121], c-myc [122], platelet-derived growth factor C [123], p57KIP [124], and PIM-3 [125]. EWS-FLI1 also regulates gene expression by modulating RNA splicing as shown by alteration of an E1A splice site and interaction with U1C [126,127]. Despite these numerous activities, the EWS-FLI1 fusion protein was shown to approach a largely unfolded conformation under native conditions [128].

### Hubness and intrinsic disorder in human diseasome

Linear regression of disorder content with respect to number of related diseases, number of related disease classes, and gene degree, shows that the correlation between disorder content and these graph-related gene features are positive and significant. The very low R^2 ^coefficient tells us that disorder content cannot be predicted from these features (which was never our intention), but that the positive correlations should be observed as trends. Two genes with the highest number of related diseases are *PAX6*, encoding a developmentally regulated transcription factor paired box protein 6 (Pax-6), which is related to 9 ophthalmological and one developmental disorder, and *TP53*, a well-studied gene encoding another transcription factor p53, that is involved in 11 different forms of cancer. Some peculiarities of the p53 structure and functions as well as a role of ID in function of this protein were already discussed (see above), whereas a brief overview of the Pax-6 protein is presented below. Pax-6 is a member of a family of developmentally regulated transcription factors that includes at least 8 members expressed in temporally and spatially restricted patterns during development and have been implicated in a number of human congenital disorders, as well as in tumorigenesis [129]. These proteins are characterized by the presence of a specific DNA-binding domain, termed the paired domain. They are highly conserved across millions of years of evolution and human *PAX-6 *gene is identical to that of axolotol [129]. CD and NMR structural analyses of the purified Pax-6 reveal that it is largely unstuctured in solution. However, upon binding to the recognition DNA sequence, the Pax-6 folds and displays CD spectroscopic evidence of significant α-helical structure [129].

A number of related disease classes and gene degree are features related to whether a gene/protein is a hub. The observed trends in predicted disorder content provide additional support for the hypothesis that hub proteins are more likely to be disordered, to accommodate the various interactions and functions they are involved with [26]. All three graph-related gene features are related to the partition of the HDN/DGN graph into one large connected component and a series of small connected components. Genes for which any of the three graph-related features is a high number belong to the large component. Since such genes are more likely to be disordered, they contribute to the difference in disorder content between large component and small components. This difference is particularly significant for genes related to metabolic diseases. More than 60% of metabolic disease genes that belong to the small components have disorder content in the 0–20% range, and further 30+% have disorder content in the 20–30% range. On the other hand, 25% metabolic disease proteins that belong to the large component have disorder content higher than 40%, which is lower when compared to other disease proteins, but substantially higher than the level of ID in metabolic disease proteins in the small component. Of note, most of metabolic disease genes in the large component are also related to disease from other classes.

The difference in disorder content between one large connected component of HDN/DGN and remaining small connected components has to be observed with caution. The connectivity of HDN/DGN is influenced heavily by small components. Only one link between a gene/disease in the large component and a disease/gene in some small components that has not yet been established, but is discovered in the future can change the partition completely, by leading to inclusion of that whole small component into the large component.

### Alternative splicing, intrinsic disorder and human genetic diseases

Prediction of intrinsic disorder in proteins encoded by genes with alternative splicing shows that AS regions have a much higher predicted disorder content than the whole protein sequences. This is in agreement with previous observations [32]. No difference was observed in disorder content for AS regions in disease and non-disease genes/proteins; the distributions were almost identical. However, alternative splicing can be observed as an important link between diseases and intrinsic disorder, as several disease classes have significantly higher fraction of genes with multiple isoforms; i.e., with AS regions. The presence of AS regions in such genes is associated with increased disorder content. Distributions of disorder content in AS regions were fairly similar across various genes, except for three classes. DEVE and NEUR have a very high fraction of highly disordered AS regions (disorder content 80–100%). This fact might be related to the functionality of proteins involved in these diseases (see above). AS regions in META genes are much less disordered than AS regions in other disease classes, just like whole META gene sequences are much less disordered than other disease genes.

### Abundance of α-MoRFs in proteins associated with human genetic diseases

IDRs frequently participate in protein-protein interactions and molecular recognitions [1,5,10,22,30,34,130]. Many IDPs and IDRs undergo disorder-to-order transitions upon binding, which is crucial for recognition, regulation, and signaling [1,4,14,28,34-37,131-133]. A recent confounding observation is that not all specific interactions between intrinsically disordered proteins and their partners are necessarily accompanied by the disorder-to-order transitions, but may somehow remain unstructured even after binding [134-138]. Nevertheless, a correlation has been established between the specific pattern in the PONDR^® ^VL-XT curve and the ability of a given short disordered regions to undergo a disorder-to-helix transition upon binding [139]. Based on these specific features, a predictor helix-forming MoRFs was recently developed [34,37]. Not all helix forming MoRF regions share these same features, and some MoRFs form β- or irregular structure rather than the α-helix [35,36]. A further complication is that MoRFs can exhibit partner-dependent structures, with at least one example morphing into helix, sheet, or irregular structure, depending on the partner [28]. Overall, therefore, these predicted MoRFs represent only fractions of the total numbers of MoRFs for each organism.

The application of the α-MoRF predictor to various datasets reveals that helix forming molecular recognition features are highly abundant in proteins associated with all human genetic diseases as well as in proteins encoded by disease genes and by all human genes, suggesting the existence of extensive interaction networks. In the HUM set, 57.9% of human genes contain α-MoRFs. In the DIS set, 54.4% of all disease-associated genes contain α-MoRFs, with significant variation between various disease classes, ranging from 26.0% in metabolic diseases and 27.3% in nutritional disorders to 73.4% in cancer and 78.6% in skeletal diseases. In most disease classes some long, highly disordered proteins have multiple predicted α-MoRF regions (Table 4) that may potentially serve as binding sites for multiple proteins. For example, DMD from CARD/MUSC (7 predicted α-MoRFs, 3771 amino acids, 54.4% disorder content); MITF from MCD (5, 598, 75.6%); DTNA from CARD (4, 767, 61.0%); EDA from DERM (4, 460, 63.9%); PLEC1 from DERM/MUSC (3, 4904, 56.6%); BRCA1 from CANC (3, 1864, 80.6%); GNAS from BONE/CANC/MCD/ENDO (3, 1323, 74.9%); OPA1 from OPHT (3, 1015, 36.1%); CD44 from HEMA (3, 807, 76.0%); COLQ from NEUR (3, 622, 76.0%); PITX2 from MCD/OPHT (3, 385, 81.0%); FAS from CANC/IMMU (3, 376, 59.3%); MXI1 from CANC (3, 320, 88.8%).

**Table 4:** Disease-related genes with multiple predicted α-MoRFs.

**SKEL (44/56 ~78.6%)**	FGFR1 (2, 901, 38.5%)
**BONE (29/44 ~65.9%)**	GNAS (3, 1323, 74.9%), COL9A1 (2, 945, 76.5%), AMELX (2, 205, 66.8%)
**DERM (55/80 ~68.8%)**	EDA (4, 460, 63.9%), PLEC1 (3, 4904, 56.6%), ADAR (2, 1226, 53.6%), SLC39A4 (2, 686, 36.3%), PVRL1 (2, 658, 37.2%)
**CANC (152/207 ~73.4%)**	BRCA1 (3, 1864, 80.6%), GNAS (3, 1323, 74.9%), FAS (3, 376, 59.3%), MXI1 (3, 320, 88.8%), ATM (2, 3056, 21.1%), DLC1 (2, 1591, 62.7%), PML (2, 1225, 63.9%), ABL1 (2, 1175, 59.2%), AR (2, 927, 60.0%), CHEK2 (2, 586, 37.2%), CASP8 (2, 548, 38.5%), PARK2 (2, 465, 37.0%), SMARCB1 (2, 385, 37.1%), SSX2 (2, 255, 77.3%)
**DEVE (36/53 ~67.9%)**	NSD1 (2, 2706, 77.3%), UBE3A (2, 882, 27.8%), PVRL1 (2, 658, 37.2%), TGIF1 (2, 424, 77.4%)
**MCD (142/209 ~67.9%)**	MITF (5, 598, 75.6%), GNAS (3, 1323, 74.9%), PITX2 (3, 385, 81.0%), NSD1 (2, 2706, 77.3%), ATRX (2, 2492, 72.2%), COL11A1 (2, 1857, 81.3%), COL18A1 (2, 1551, 74.4%), L1CAM (2, 1257, 27.0%), USH1C (2, 926, 58.4%), FGFR1 (2, 901, 38.5%), HPS4 (2, 783, 42.9%), KCNQ1 (2, 718, 42.3%), PVRL1 (2, 658, 37.2%), DTNBP1 (2, 383, 81.7%)
**CARD (53/96 ~55.2%)**	DMD (7, 3771, 54.4%), DTNA (4, 767, 61.0%), KCNH2 (2, 1283, 46.0%), KCNQ1 (2, 718, 42.3%), EYA4 (2, 665, 61.7%), TPM1 (2, 443, 100.0%)
**MUSC (45/68 ~66.2%)**	DMD (7, 3771, 54.4%), PLEC1 (3, 4904, 56.6%), COL6A3 (2, 3177, 28.6%), AR (2, 927, 60.0%), CHAT (2, 748, 34.1%), TPM3 (2, 378, 97.6%)
**IMMU (59/115 ~51.3%)**	FAS (3, 376, 59.3%), ATM (2, 3056, 21.1%), PTPRC (2, 1307, 42.2%), CASP8 (2, 548, 38.5%), PARK2 (2, 465, 37.0%), UNG (2, 348, 44.5%)
**OPHT (68/120 ~56.7%)**	OPA1 (3, 1015, 36.1%), PITX2 (3, 385, 81.0%), PIP5K3 (2, 2108, 52.0%), EYA1 (2, 600, 60.5%)
**CTD (32/51 ~62.7%)**	--
**ENDO (54/96 ~56.3%)**	GNAS (3, 1323, 74.9%), AR (2, 927, 60.0%), HNF4A (2, 531, 51.6%), GCK (2, 495, 35.6%)
**NEUR (154/254 ~60.6%)**	COLQ (3, 622, 76.0%), PTPRC (2, 1307, 42.2%), L1CAM (2, 1257, 27.0%), KCNQ2 (2, 892, 55.5%), FOXP2 (2, 740, 78.9%), MTMR2 (2, 643, 31.6%), SPAST (2, 616, 51.1%), EYA1 (2, 600, 60.5%), NR4A2 (2, 599, 55.8%), EIF2B4 (2, 555, 47.2%), CACNB4 (2, 538, 60.0%), OPRM1 (2, 492, 34.6%), CCM2 (2, 475, 54.1%), PARK2 (2, 465, 37.0%), DCX (2, 446, 57.8%), DRD2 (2, 443, 39.1%), PNKD (2, 440, 39.5%), ATXN3 (2, 370, 61.6%), FGF14 (2, 316, 46.2%)
**PSYC (18/30 ~60.0%)**	--
**ENT (26/44 ~59.1%)**	OTOF (2, 2100, 33.9%), USH1C (2, 926, 58.4%), KCNQ4 (2, 695, 41.3%), EYA4 (2, 665, 61.7%)
**RESP (12/34 ~35.3%)**	GDNF (2, 230, 51.3%)
**RENA (35/58 ~60.3%)**	--
**HEMA (58/146 ~39.7%)**	CD44 (3, 807, 76.0%), ATRX (2, 2492, 72.2%), ANK1 (2, 2001, 40.9%), ADAMTS13 (2, 1497, 48.6%), EPB41 (2, 850, 66.2%), AMPD3 (2, 781, 36.9%), IGLL1 (2, 228, 86.4%)
**NUTR (6/22 ~27.3%)**	--
**GI (16/34 ~47.1%)**	GDNF (2, 230, 51.3%)
**UNCL (12/29 ~41.4%)**	--
**META (75/289 ~26.0%)**	GCK (2, 495, 35.6%), HFE2 (2, 426, 42.0%)

The class identifier is followed by the number of genes with predicted α-MoRFs, the total number of genes, and the fraction of genes with predicted α-MoRFs in that class. This is followed by the list of genes with multiple (>= 2) predicted α-MoRFs. The numbers following each gene symbol are the number of predicted α-MoRFs, the number of amino acids and the disorder content (note that in the case of alternative splicing the number of amino acids includes all exons in a gene and may be larger than the lengths of individual isoforms).

Interestingly, fractions of proteins with predicted α-MoRF regions were highly correlated with the content of predicted disorder in a given dataset (correlation coefficient is ~0.89). This suggests that the major function of IDRs in the proteins from analyzed datasets is protein-protein interaction. α-MoRFs, being disordered in the unbound state and gaining α-helical structure upon interaction with binding partners, suit ideally this function. In fact, it has been proposed that that the involvement of IDRs in protein-protein interactions have several advantages [27], including: (i) Decoupled specificity and strength of binding (high-specificity-low-affinity interactions); (ii) Increased speed of interaction due to greater capture radius and the ability to spatially search interaction space; (iii) Efficient regulation via rapid degradation; (iv) Increased interaction (surface) area per residue; (v) Strengthened encounter complex (less stringent spatial orientation requirements); (vi) A single disordered region may bind to several structurally diverse partners; (vii) Many (structured) proteins may bind a single disordered region; (viii) Less sterically restricted to allow elongation of binding area; (ix) Efficient regulation via posttranslational modification; (x) Ease of regulation/redirection by alternative splicing; (xi) Overlapping binding sites due to extended linear conformation; (xii) High evolutionary rate; (xiii) Flexibility that allows masking (or not) of interaction sites or allow interaction between bound partners. Many of these features are specific properties of α-MoRFs.

### Abundance of α-MoRFs in alternative spliced regions of proteins from human diseasome

Interestingly, our analysis revealed that α-MoRFs are abundantly present in alternatively spliced regions of proteins from some human genetic diseases. This observation is very important as it sheds some light on the potential functional repertoire of alternatively spliced regions. In several diseases, these regions play a crucial role in protein-protein interaction, as they are enriched in molecular recognition features.

## Concluding remarks

Intrinsically disordered proteins are highly abundant in nature. Although they lack stable tertiary and/or secondary structure under physiological conditions *in vitro*, IDPs carry out a number of crucial biological functions, being involved in regulation, recognition, signaling and control. The functional repertoire of IDPs complements the functions of ordered proteins. Earlier studies revealed that many IDPs are associated with various human diseases, including cancer, cardiovascular disease, amyloidoses, neurodegenerative diseases, diabetes and others, emphasizing the existence of intriguing interconnections between IDPs, cell signaling and human diseases. Based on these observations, the "disorder in disorders" or D^2 ^concept was introduced.

Here, a large-scale analysis of the abundance of intrinsic disorder in human genetic diseases joined into the human diseasome [47] was performed. This analysis uncovered an unfoldome (an IDP-containing subset of a proteome) associated with human genetic diseases and revealed several interesting peculiarities. Particularly, we are showing here that proteins associated with various human genetic diseases are enriched in intrinsic disorder with the IDP content being markedly different for different genetic diseases. The diseasome possesses a high level of MoRFs, whose abundance correlates with the intrinsic disorder level. Alternative splicing is commonly present in several genetic diseases. Alternatively spliced regions in corresponding proteins are predicted to be highly disordered and in some diseases contain a significant number of MoRFs. The various diseasome graph-related properties are correlated with the levels of intrinsic disorder (hub proteins are generally more disordered). These data were used to build the unfoldome for the diseaseome.

## Competing interests

The authors declare that they have no competing interests.

## Authors' contributions

VNU was involved in design and planning of all the experiments, contributed to the manuscript writing, revised the final version and headed the project. UM performed the computational analysis, designed figures and drafted the manuscript. CJO performed the computational analysis and contributed to the manuscript writing. ZO and AKD were involved in design and planning of all the experiments and contributed to the manuscript writing. All authors have read and approved the final manuscript.
